# Inflammatory Biomarkers Predictive of Psychopathology in Children With Physical Illness

**DOI:** 10.1002/brb3.70726

**Published:** 2025-08-04

**Authors:** Mark A. Ferro, Christy K. Y. Chan, Fasih A. Rahman, Joe Quadrilatero, Brian W. Timmons

**Affiliations:** ^1^ School of Public Health Sciences University of Waterloo Waterloo Ontario Canada; ^2^ Department of Kinesiology and Health Sciences University of Waterloo Waterloo Ontario Canada; ^3^ Department of Pediatrics McMaster University Hamilton Ontario Canada

**Keywords:** adolescent, chronic disease, cytokine, inflammation, psychiatric disorder

## Abstract

**Background:**

While evidence shows that children with chronic physical illness are at increased risk for psychopathology, the causal mechanisms linking physical and mental illness early in life remain unconfirmed.

**Objective:**

Given a lack of longitudinal studies investigating inflammatory responses in the context of child psychopathology, and none that specifically sample children with chronic physical illness, this study evaluated associations between inflammatory biomarkers and psychopathology in children with chronic physical illness over a 48‐month period.

**Methods:**

Data come from 128 children enrolled in the Multimorbidity in Children and Youth across the Life‐course (MY LIFE) study who provided baseline dried blood samples for biomarker analysis and completed the 48‐month assessment (mean age 11.0 years, 50.0% male). Psychopathology was measured using parent and child reports on the Emotional Behavioural Scales. Dried blood samples were assayed using the Bio‐Plex 200 system. Linear mixed models, adjusted for age and sex, estimated associations between inflammatory biomarkers and child psychopathology over time.

**Results:**

No differences in biomarker concentrations across physical illnesses were found, except for interleukin (IL)‐1β, which showed lower levels in the neurological subgroup (*χ* ^2^ = 13.27, *p* = 0.04). Elevated levels of granulocyte colony‐stimulating factor (G‐CSF) were associated with higher parent‐reported internalizing symptoms (*β* = 0.09 [0.04]), whereas elevated granulocyte‐macrophage colony‐stimulating factor (GM‐CSF) was associated with child‐reported (*β* = 1.83 [0.46]) and parent‐reported total symptoms (*β* = 1.52 [0.69]), as well as child‐reported internalizing (*β* = 1.35 [0.28]) and parent‐reported externalizing symptoms (*β* = 0.72 [0.30]). Elevated IL‐6 was associated with lower child‐reported total symptoms (*β* = −0.87 [0.27]) and parent‐reported externalizing symptoms (*β* = −0.35 [0.16]) over time.

**Conclusion:**

Findings are not definitive evidence of a causal mechanism for physical–mental comorbidity in children but are consistent with reports in other populations. The predictive power of G‐CSF, GM‐CSF, and IL‐6 supports the need for broader blood panels and routine mental health assessment, particularly for children on treatments that disrupt regulatory inflammatory pathways.

## Graphical abstract

Elevated levels of granulocyte colony‐stimulating factor (G‐CSF) were associated with higher parent‐reported internalizing symptoms (β = 0.09 [0.04]); whereas, elevated granulocyte‐macrophage colony‐stimulating factor (GM‐CSF) was associated with child‐ (β = 1.83 [0.46]) and parent‐reported total symptoms (β = 1.52 [0.69]), as well as child‐reported internalizing (β = 1.35 [0.28]) and parent‐reported externalizing symptoms (β = 0.72 [0.30]). Elevated IL‐6 was associated with lower child‐reported total symptoms (β = ‐0.87 [0.27]) and parent‐reported externalizing symptoms (β = ‐0.35 [0.16]) over time.

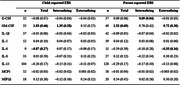

## 1 Introduction

Accumulating evidence points to an increased propensity for psychopathology among children with chronic physical illness compared to their healthy counterparts; however, causal mechanisms remain unconfirmed (Leyenaar et al. 2024). Research suggests shared immunological or inflammatory responses for physical and mental illnesses, but these findings are often based on cross‐sectional data (Howe and Lynch 2022). One prospective study found that cord serum levels of tumor necrosis factor‐α (TNF‐α) were inversely associated with mood dysregulation trajectories when children were between 3 and 8 years of age (Herbein et al. 2024). However, that study did not purposefully sample children with physical illness—a group with inherently elevated systemic inflammation. We address this knowledge gap by evaluating associations between inflammatory biomarkers and psychopathology over 48 months in children with chronic physical illness.

## 2 Methods

Data come from Multimorbidity in Children and Youth across the Life‐course (MY LIFE) (Ferro et al. 2022), an ongoing prospective study of psychopathology among children aged 2–16 years diagnosed by a health professional with chronic physical illness (e.g., asthma, diabetes, juvenile arthritis) recruited from the outpatient clinics at a pediatric hospital in Canada. Methodological details of MY LIFE and characteristics of the cohort have been previously described (Ferro et al. 2022). MY LIFE was approved by the Hamilton Integrated Research Ethics and Waterloo Human Research Ethics Boards. Of the 263 children enrolled at the outset of MY LIFE, 235 (89.4%) completed the 48‐month follow‐up, and 128 (48.7%) children provided blood samples for biomarker analysis.

The Emotional Behavioural Scales (EBS) measured Diagnostic and Statistical Manual of Mental Disorders (DSM)‐5‐aligned child psychopathology at five assessments (enrollment and 6, 12, 24, and 48 months). Responses to EBS items are anchored to a 3‐point Likert scale and are summed to generate scores for internalizing, externalizing, and total symptoms. Higher scores on the EBS indicate more severe psychopathology. Internal consistency of the EBS outcomes in this sample was α > 0.80.

Inflammatory biomarkers were assayed from dried blood spots using a human cytokine panel (Bio‐Rad, #M5000031YV). The biomarkers included in the panel and the proportion of samples with a signal (i.e., above the lower limit of quantification) were granulocyte colony‐stimulating factor (G‐CSF; 28.9%), elevated granulocyte‐macrophage colony‐stimulating factor (GM‐CSF; 31.3%), interferon‐γ (0.0%), interleukin‐1β (IL‐1β; 32.8%), IL‐2 (0.0%), IL‐4 (0.0%), IL‐5 (46.1%), IL‐6 (8.6%), IL‐7 (0.0%), IL‐8 (28.9%), IL‐10 (0.0%), IL‐12 (1.6%), IL‐13 (100.0%), IL‐17 (0.8%), monocyte chemoattractant protein (MCP1; 45.3%), macrophage inflammatory protein‐1β (MIP1β; 15.6%), and TNF‐α (0.0%). Data acquisition was performed on a Bio‐Plex 200 system and displayed as fluorescence intensity, which was converted to a concentration in picograms per milliliter. Only biomarkers with a signal proportion of ≥5.0% were analyzed.

One‐way analysis of variance (ANOVA) tested for differences in EBS scores across physical illnesses classified by the International Classification of Diseases, Functioning, and Disability, Tenth Revision (ICD‐10) and repeated‐measures ANOVA for differences over time. Because of deviations from normality, the nonparametric Kruskal–Wallis test examined differences in biomarker concentrations across ICD‐10 classifications of physical illnesses (i.e., dermatological, endocrine, gastroenterological, hematological, neurological, respiratory, rheumatological). Linear mixed models with random intercepts and slopes, adjusted for age and sex, estimated associations between inflammatory biomarkers measured at study enrollment and child psychopathology over 48 months. Hypothesis tests were two‐sided with α = 0.05. Data were analyzed using SPSS 28.

## 3 Results

The mean age of children at enrollment was 11.0 (4.1) years, and 64 (50.0%) were male. Mean total, internalizing, and externalizing child‐ and parent‐reported EBS scores at enrollment were 20.8 (13.2), 12.8 (8.8), and 8.0 (5.7), and 18.0 (14.1), 10.5 (9.0), and 7.6 (6.7), respectively. Child‐ and parent‐reported total, internalizing, and externalizing EBS scores at 48 months were 22.5 (16.2), 14.8 (11.4), and 7.7 (5.9), and 15.4 (13.3), 9.1 (8.7), and 6.2 (6.5), respectively. Significant decreases in EBS over time were found for parent reports of total (*F* = 2.72, *p* = 0.04) and externalizing symptoms (*F* = 3.31, *p* = 0.02). No other differences in EBS scores over time were found. There were no differences in EBS scores across classifications of physical illness (range *F* = 0.03–1.30, *p* > 0.05 for all).

There was no evidence of differences in biomarker concentrations across classifications of physical illness (range *χ* ^2^ = 4.26–10.50, *p* > 0.05 for all), except for IL‐1β, which showed significantly lower levels among children in the neurological subgroup compared to all other subgroups (*χ* ^2^ = 13.27, *p* = 0.04). Table 1 shows that elevated levels of G‐CSF were associated with higher parent‐reported internalizing symptoms (*β* = 0.09 [0.01, 0.17]), whereas elevated GM‐CSF was associated with child‐reported (*β* = 1.83 [0.93, 2.73]) and parent‐reported total symptoms (*β* = 1.52 [0.17, 2.87]), as well as child‐reported internalizing (*β* = 1.35 [0.80, 1.90]) and parent‐reported externalizing symptoms (*β* = 0.72 [0.13, 1.31]). In contrast, elevated IL‐6 was associated with lower child‐reported total symptoms (*β* = −0.87 [−1.40, −0.34]) and parent‐reported externalizing symptoms (*β* = −0.35 [−0.66, −0.04]) over time. No other associations were found.

**TABLE 1:** Linear mixed models of child‐ and parent‐reported psychopathology over 48 months.

	Child‐reported EBS				Parent‐reported EBS			
	*n*	Total	Internalizing	Externalizing	*n*	Total	Internalizing	Externalizing
G‐CSF	32	−0.08 (0.07)	−0.04 (0.04)	−0.04 (0.03)	37	0.09 (0.06)	**0.09 (0.04)**	−0.01 (0.03)
GM‐CSF	35	**1.83 (0.46)**	**1.35 (0.28)**	0.52 (0.27)	40	**1.52 (0.69)**	0.78 (0.42)	**0.72 (0.30)**
IL‐1β	37	−0.05 (0.06)	−0.03 (0.04)	−0.02 (0.03)	42	−0.09 (0.05)	−0.07 (0.04)	−0.02 (0.02)
IL‐5	52	0.04 (0.10)	0.04 (0.07)	0.03 (0.05)	59	0.04 (0.12)	0.03 (0.08)	0.01 (0.06)
IL‐6	9	**−0.87 (0.27)**	0.07 (0.17)	−0.09 (0.17)	11	−0.59 (0.39)	−0.18 (0.28)	**−0.35 (0.16)**
IL‐8	33	0.05 (0.50)	−0.07 (0.31)	0.11 (0.23)	37	0.12 (0.52)	−0.22 (0.34)	0.30 (0.23)
IL‐13	104	−0.26 (0.17)	−0.15 (0.11)	−0.11 (0.07)	128	−0.29 (0.17)	−0.17 (0.10)	−0.13 (0.08)
MCP1	52	−0.02 (0.03)	−0.02 (0.02)	0.001 (0.02)	58	−0.01 (0.04)	−0.01 (0.02)	−0.003 (0.02)
MIP1β	18	0.12 (0.26)	−0.12 (0.16)	0.24 (0.12)	20	0.34 (0.45)	0.02 (0.26)	0.30 (0.20)

*Note*: Data are *β* (standard error). Those in bold are statistically significant at α = 0.05. Fixed effects of random intercepts and slopes (linear and quadratic time) were included in the models. Models were adjusted for child age and sex. Continuous variables were grand‐mean centered. Inflammatory markers were rescaled to increase the interpretability of the models such that unit increases corresponded to 0.01 pg/mL, except for GM‐CSF, for which unit increases corresponded to 0.001 pg/mL.

Abbreviations: EBS, Emotional Behavioural Scales; G‐CSF, granulocyte colony‐stimulating factor; GM‐CSF, granulocyte‐macrophage colony‐stimulating factor; IL, interleukin; MCP, monocyte chemoattractant protein; MIP, macrophage inflammatory protein.

## 4 Discussion

Small sample sizes and concerns for multiple testing prevented the investigation of specific psychopathologies and the computation of age‐ and sex‐stratified models. Information on pharmacotherapies that target inflammation, psychotropics prescribed for child emotional or behavioral problems, or psychosocial interventions was not available in MY LIFE; thus, estimates of association may be underestimated. Consistent with previous research (Buytaert et al. 2024), several biomarkers were below the lower limit of quantification and could not be modeled.

While the associations reported are not definitive evidence of a causal mechanism for physical–mental comorbidity in children, findings are consistent with positive (G‐CSF, GM‐CSF) and inverse (IL‐6) associations between inflammatory biomarkers and psychopathology in other child populations (Howe and Lynch 2022) and have relevant clinical implications. For instance, broader panels of inflammatory biomarkers when ordering blood work, especially early in the course of illness, should be considered to help identify children at increased risk for physical–mental comorbidity and reduce referral times for early mental health intervention within integrated pediatric care. The longer term predictive power of G‐CSF, GM‐CSF, and IL‐6 supports the need for routine mental health assessment, particularly for children on treatments that disrupt regulatory inflammatory pathways. Research that aims to establish inflammatory biomarker profiles of children with physical illness with reference to pediatric norms (data that remain scarce) would make an important contribution to understanding the intersection of physical and mental illness early in life.

## Author Contributions

Mark A. Ferro conceptualized and designed the study, acquired funding, coordinated and supervised data collection, drafted the initial manuscript, and critically reviewed and revised the manuscript. Christy K. Y. Chan and Fashi A. Rahman carried out the initial analyses and critically reviewed and revised the manuscript. Joe Quadrilatero and Brian W. Timmons conceptualized and designed the study, acquired funding, and critically reviewed and revised the manuscript for important intellectual content. All authors approved the final manuscript as submitted and agreed to be accountable for all aspects of the work.

## Ethics Statement

This study was approved by the Hamilton Integrated Research Ethics (2797) and Waterloo Human Research Ethics Boards (31010).

## Consent

Informed consent from all parents on behalf of themselves and their children was obtained. Children aged 16 years also provided informed consent. In the presence of their parents, assent was obtained from children aged 7–15 years. Parents consented on behalf of all children who were ≤6 years of age.

## Conflicts of Interest

The authors declare no conflicts of interest.

## Peer Review

The peer review history for this article is available at https://publons.com/publon/10.1002/brb3.70726

## Data Availability

Ethical approval was not obtained for the sharing of study data. Individual requests for data access can be made to the corresponding author and will be considered by the study team on a case‐by‐case basis.
